# Duodenal perforation following right nephrectomy in a paraplegic patient with pyonephrosis: a case report and clinical insights

**DOI:** 10.3389/fmed.2026.1745528

**Published:** 2026-04-01

**Authors:** Jun Li, Xiong Wang, Xiaoshuang Tang

**Affiliations:** 1 Ankang Central Hospital, Ankang, Shaanxi, China; 2 The Ankang Hospital for Maternity and Child Health, Ankang, Shaanxi, China; 3 Department of Urology, The Second Affiliated Hospital of Xi’an Jiaotong University, Xi’an, China

**Keywords:** duodenal perforation, multidisciplinary team collaboration, paraplegia, right nephrectomy, type 2 diabetes mellitus

## Abstract

Duodenal perforation is a life-threatening acute complication with high morbidity and mortality, particularly among patients with multiple comorbidities. We present the case of a 46-year-old Asian male with a history of paraplegia and type 2 diabetes mellitus who developed duodenal perforation following right nephrectomy for non-functional kidney complicated by perirenal abscess and lumbar sinus tract infection. The patient sustained a thoracolumbar fracture and spinal cord injury due to blunt trauma 6 years prior, resulting in permanent lower limb paraplegia. Preoperative imaging confirmed right renal non-function, and sequential interventions including perirenal abscess aspiration, ureteral stenting, and nephrectomy were performed. Duodenal perforation (4 mm in diameter) was identified via gastroscopy on postoperative day 6, initially managed with endoscopic clipping, and subsequent radical surgery (subtotal gastrectomy + Roux-en-Y gastrojejunostomy + cholecystolithotomy + cholecystostomy) was required due to persistent leakage. Multidisciplinary team (MDT) collaboration, targeted antibiotic therapy based on pharmacokinetic/pharmacodynamic (PK/PD) principles, and staged nutritional support were implemented. The patient achieved clinical cure after 3 months of comprehensive management. This case highlights the importance of early diagnosis using computed tomography (CT) and endoscopy, individualized surgical strategies, and MDT collaboration in managing postoperative duodenal perforation in high-risk patients with paraplegia and diabetes.

## Introduction

Duodenal perforation is one of the most severe acute complications of the duodenum, characterized by acute abdomen, high mortality, and distinct clinical features, etiological profiles, and therapeutic strategies (1). Typical manifestations include sudden severe abdominal pain, signs of peritoneal irritation (muscle rigidity, rebound tenderness), and systemic infectious symptoms such as fever and leukocytosis. However, approximately 30% of cases involve the retroperitoneal segment, leading to atypical symptoms that may mimic other acute abdominal conditions and result in misdiagnosis. CT is the cornerstone of diagnosis, enabling clear visualization of free gas, local effusion, or retroperitoneal pneumatosis. Etiologically, peptic ulcers account for over 50% of cases, followed by iatrogenic injuries (e.g., sphincterotomy during endoscopic retrograde cholangiopancreatography [ERCP], endoscopic submucosal dissection [ESD]) (2, 3). Trauma, foreign body ingestion, malignant tumors, and acute pancreatitis are also important predisposing factors. Notably, although duodenal diverticula are present in 20% of the population, diverticular perforation is extremely rare (only 162 cases reported globally) with a mortality rate as high as 30% (4, 5).

Therapeutic strategies for duodenal perforation require individualization: limited perforations (e.g., Stapfer type I/II ERCP-related perforations) can be managed conservatively with fasting, gastrointestinal decompression, and antibiotics (6). Septicemia, diffuse peritonitis, or failed conservative treatment necessitates emergency surgery, including Graham omental patch repair and diverticulectomy. For retroperitoneal perforations, endoscopic negative pressure therapy may reduce surgical risks. Complex cases require MDT collaboration involving surgery, endoscopy, and critical care. Prognosis depends on timely diagnosis; delayed intervention significantly increases mortality—with mortality rates of approximately 10% for ERCP-related perforations and 30% for diverticular perforations (4). Pediatric cases are prone to misdiagnosis due to occult symptoms; for those with retroperitoneal perforation or complex fistulas, T-tube drainage may improve surgical outcomes (7, 8).

Patients with comorbidities such as paraplegia and diabetes face higher risks of postoperative complications and infectious sequelae, posing significant challenges to clinical management. This report details the complex clinical course of a paraplegic patient with diabetes who developed duodenal perforation after nephrectomy, from 2024 to July 2025, and discusses key clinical insights to inform the management of similar high-risk cases.

## Case presentation

### Clinical history

A 46-year-old Asian male with permanent lower limb paraplegia (resulting from a thoracolumbar spinal cord injury in 2019) and poorly controlled type 2 diabetes mellitus (diagnosed in 2024) was admitted in March 2025 for definitive surgical management of a refractory right lumbar sinus tract present for 1 year.

His clinical course had been progressive. In 2023, spontaneous ulceration with purulent exudation developed in the right lumbar region; local wound care failed, requiring repeated debridement and negative pressure therapy at our hospital for temporary relief. By 2024, the ulcer had evolved into a chronic infected sinus tract. Sequential interventions that year—including debridement with closed negative pressure aspiration (February 21 and March 8) and perirenal puncture drainage (April 3)—provided only partial symptomatic relief, and the patient discharged himself against medical advice. Subsequent evaluations at two tertiary hospitals in 2024 confirmed a non-functional right kidney (renal scintigraphy, May 21), perirenal abscess, and hydronephrosis; nephrectomy was recommended but declined. He was readmitted to our hospital on July 4, 2024, for ureteroscopic holmium laser lithotripsy and double-J stent placement, and again on January 9, 2025, due to increased exudation (ultrasound revealed renal calculi and hydronephrosis). However, symptoms recurred in February 2025, prompting his final admission in March 2025 for definitive surgery. Due to thoracic spinal cord injury-induced paraplegia, the patient had long-standing neurogenic gastrointestinal dysfunction. A gastroenterology consultation on March 26, 2025, confirmed the diagnosis of “gastroparesis syndrome/duodenal stasis.

### Examination findings

Physical examination revealed a 0.3 × 0.2 cm right lumbar wound with exudate, a subcutaneous sinus tract (>8 × 2 cm) without bony involvement, surrounding erythema (non-tender due to spinal cord injury), extensive pigmentation, and surgical scars. Lower limb paraplegia persisted with inguinal-level hypoesthesia and increased muscle tone.

**Figure 1:**
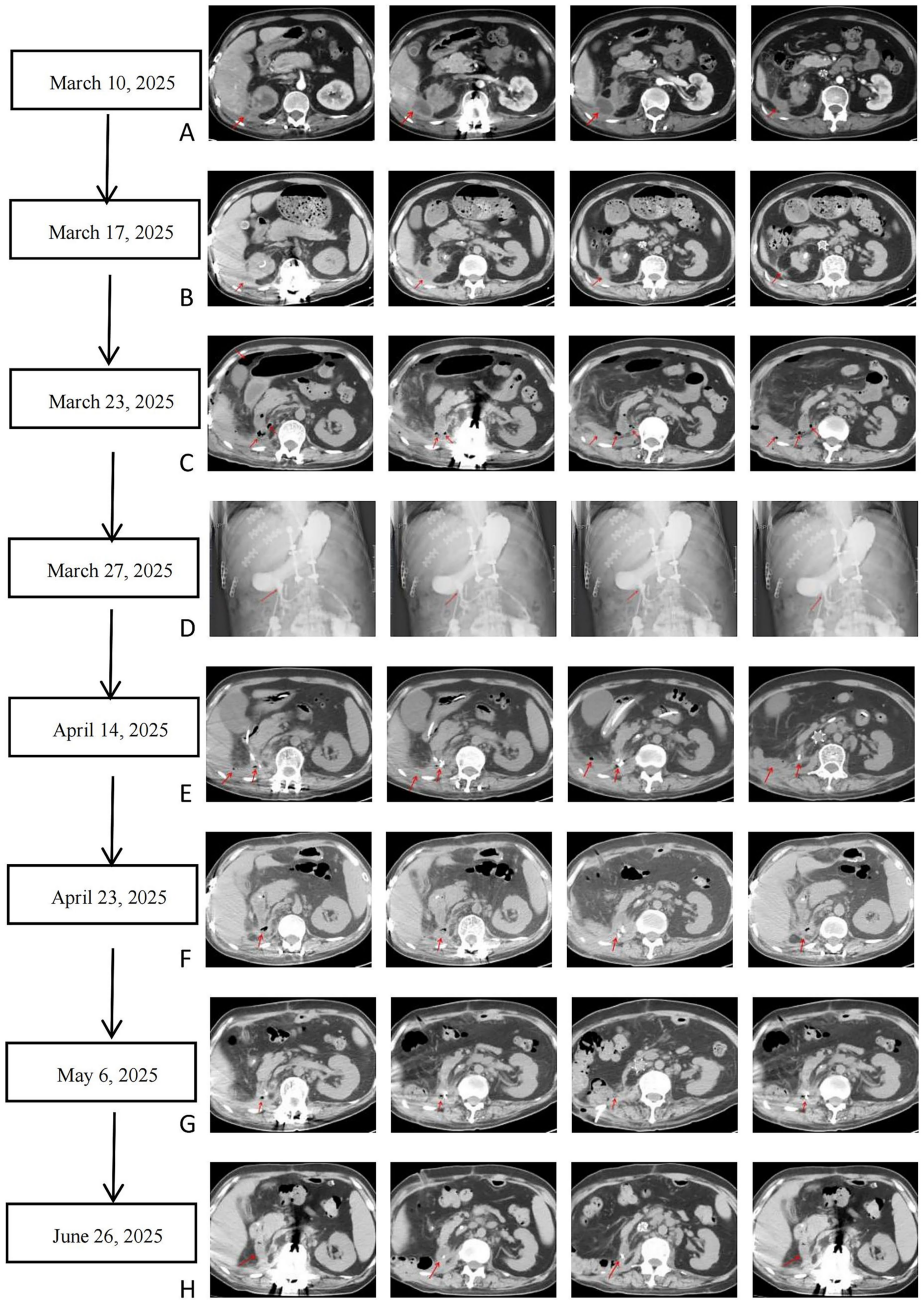
Dynamic changes of abdominal and pelvic CT images. Subfigures **(A–H)** show sequential CT findings (comparisons to prior scans): **(A,B)** Pre-nephrectomy: stable right renal calculi, double “J” stent, improved perirenal exudation (March vs. January). **(C)** Post-nephrectomy: right kidney absent, retroperitoneal drainage tube, left mild hydronephrosis. **(D)** Duodenal fistula: contrast extravasation from duodenum to abdominal cavity. **(E–H)** Post-fistula treatment: absorbed intra-abdominal gas, reduced sinus tract/encapsulated effusion, removed lumbodorsal catheter, improved surgical area fluid (March–May).

Laboratory and imaging results were key: baseline renal function was preserved (March 12, 2025: urea 6.59 mmol/L, creatinine 76.3 μmol/L, uric acid 426.4 μmol/L, eGFR 103.84 mL/(min·1.73m^2^)). Wound exudate (March 1, 2025) and perirenal aspirate (March 19, 2025, 17 mL purulent fluid) cultured *Escherichia coli* and *Proteus mirabilis*, confirming persistent infection. Postoperative drainage cultures (April 2025) identified multidrug-resistant *Enterococcus faecium* (April 9), recurrent *E. coli* (April 21), and later mixed infection with *Stenotrophomonas maltophilia* and *Candida albicans*. Serial CT scans (April 13 and 23, 2025) showed right nephrectomy changes, resolved intra-abdominal pneumatosis, reduced right iliac vein-adjacent encapsulated effusion, and a prominent lumbar sinus tract (Figure 1).

### Treatment and outcomes

**Figure 2:**
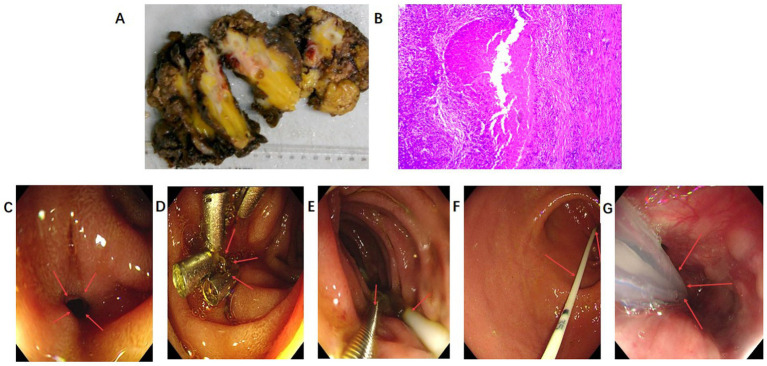
Key nodes of surgical specimens, pathological results, and duodenal endoscopic intervention. **(A)** (Postoperative specimen): right nephrectomy specimen (March 21, 2025): non-functional right kidney, perirenal fat capsule, sinus tract tissue (fixed in 10% neutral buffered formalin). **(B)** (Pathological results): diagnosis: infectious interstitial nephritis involving perirenal tissue (abscess, granulomatous inflammation, fibrosis); skin focal suppurative inflammation. IHC: No neoplasia (Ki-67 ≈ 10%, CD68+). Special staining: no obvious pathogens (mycobacteria not fully excluded). **(C–G)** (Endoscopy, March 27): 0.4 cm duodenal fistula (bulb-descending junction) closed with 4 titanium clips; feeding tube fixed (105 cm at nasal alar) + gastrointestinal decompression.

Preoperative management, guided by a multidisciplinary team (MDT), included intravenous meropenem (1 g q8h, March 13–April 2), transurethral ureteral stenting (March 17), and ultrasound-guided perirenal abscess aspiration (March 19). On March 21, 2025, the patient underwent right nephrectomy, perirenal abscess drainage, sinus tract debridement, and adhesiolysis via a 12 cm right subcostal incision under general anesthesia. Intraoperative findings included dense adhesions between the atrophic right kidney, duodenum, and ascending colon (with chronic inflammatory ossification), dissected sequentially with ultrasonic scalpel and electrocoagulation to preserve intestinal integrity; the procedure was uneventful (Figure 2).

Postoperatively, abnormal retroperitoneal drainage led to gastroscopic diagnosis of a 4 mm duodenal bulb perforation (March 27), initially managed with endoscopic clipping. Persistent leakage (1,520 mL coffee-ground fluid, April 13–14, 1770 mL, April 14–15) necessitated urgent reoperation at 16:45 on April 15: subtotal gastrectomy with Roux-en-Y gastrojejunostomy, cholecystolithotomy (1.5 cm stone), and cholecystostomy via a 12 cm midline incision (Supplementary Figure 1).

Intraoperatively, we found turbid bile-stained fluid in the retroperitoneum and lesser sac, with edematous, adherent tissues. The duodenal perforation (originally clipped) had partially dehisced, with an unhealed 8 × 10 mm defect on the posterior wall, 3 cm from pylorus and 2.5 cm from the ampulla. The edges were friable and ischemic, making local repair impossible. Given the poor tissue quality and duodenal dysmotility, we proceeded with subtotal gastrectomy and Roux-en-Y reconstruction to definitively exclude the diseased segment.

Serial postoperative drainage cultures revealed evolving microbiology: multidrug-resistant *Enterococcus faecium* (April 9), recurrent *E. coli* (April 21), and subsequent mixed infection with *Stenotrophomonas maltophilia* and *Candida albicans* (late April). Tuberculosis was systematically excluded through negative interferon-gamma release assay 3 months prior, absence of tuberculosis history or exposure, and repeatedly negative *Mycobacterium tuberculosis* cultures from sputum and urine starting March 23, 2025. The prompt resolution of fever and inflammatory markers with targeted antibacterial therapy further supported bacterial etiology. Based on microbiological results and susceptibility testing, antibiotic regimens were sequentially adjusted: meropenem (March 13–April 2) → cefoperazone-sulbactam (from April 3) → vancomycin (from April 15) → imipenem combined with caspofungin (from April 20).

**Figure 3:**
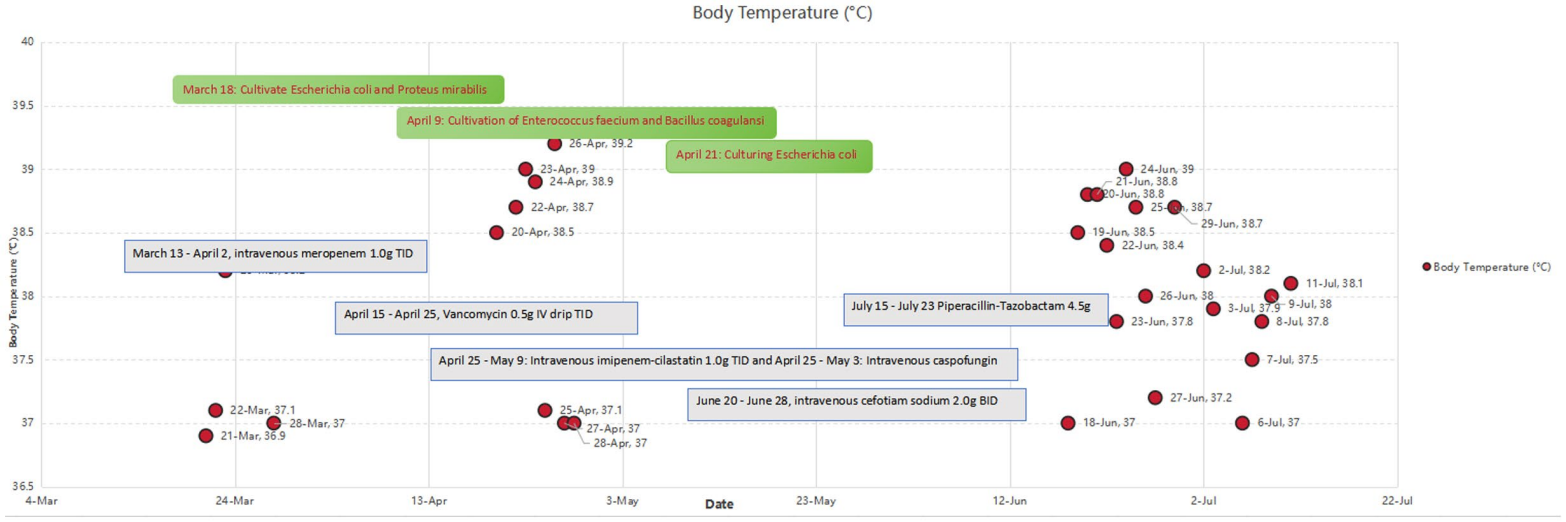
Correlation between body temperature (≥37.0 °C), pathogenic detection, and antibiotic administration. Abscissa: timeline (April 3–July 22, 2025); Ordinate: body temperature (°C, only ≥37.0 °C shown). Red asterisks: pathogen detection (April 9: *Enterococcus faecium/Bacillus coagulans*; April 21: *E. coli*; late April: mixed infection). Blue shading: antibiotic regimens (e.g., March 13–April 2: meropenem 1.0 g IV TID). Temperature peaks (e.g., 39.4 °C, April 21) correspond to infection recurrence; stabilization post-May indicates effective control.

**Figure 4:**
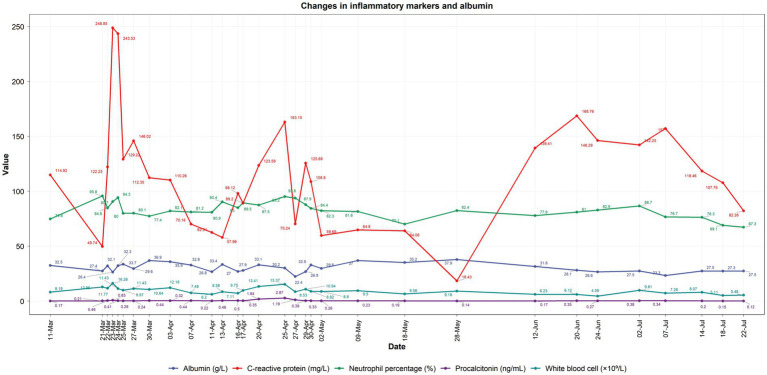
Dynamic changes in inflammatory markers and albumin levels during treatment. Abscissa: treatment timeline (March 11–July 22, 2025); Ordinates: detection indicators with units—procalcitonin (ng/mL), white blood cell count (×10^9^/L), albumin (g/L), neutrophil cell percentage (%), C-reactive protein (mg/L). Curves reflect infection control (e.g., decreasing C-reactive protein) and nutritional status improvement (stable albumin) during 3-month treatment, consistent with clinical interventions (antibiotic adjustment, nutritional support). Data from daily laboratory tests.

Postoperative rehabilitation (April–July 2025) included fluid-electrolyte correction, staged nutrition (total parenteral nutrition: 2000 kcal/day, 80 g/day protein; transitioning to enteral nutrition at 50 mL/h from April 17), insulin pump glycemic control, and targeted antibiotics (meropenem → cefoperazone-sulbactam → vancomycin → imipenem + caspofungin). Intermittent low-grade fever (max 38.9 °C) resolved; retroperitoneal drainage ceased by July 2, with tube removal on July 9. Inflammatory markers normalized by July 18 (procalcitonin \ < 0.2 ng/mL, C-reactive protein \ < 10 mg/L), and sacral grade II pressure ulcers healed with topical therapy. Postoperatively, the patient experienced recurrent acid reflux and vomiting—clinical manifestations consistent with the preexisting neurogenic dysmotility. These symptoms improved with prokinetic agents and acupuncture (initiated May 11), validating the pathological mechanism of reduced gastrointestinal motility secondary to spinal cord injury. Three-month follow-up (July 2025) confirmed clinical cure: no fever, hematuria, or leakage, with resolved nausea and abdominal distension (Figures 3, 4).

The key clinical events are summarized in a timeline (Supplementary Figure 2).

Throughout this arduous clinical course, the patient maintained unwavering trust and open communication with the multidisciplinary team. Despite enduring severe postoperative perforation and two major surgeries, he demonstrated remarkable resilience. Upon discharge, he reflected: “It was a difficult journey, but I always felt the team stood by me.” At three-month follow-up, the right lumbar sinus tract had healed completely, gastrointestinal function was stable (occasional mild bloating only), and he expressed profound satisfaction with his recovery.

## Discussion

### Treatment of duodenal ulcer perforation: stepwise progression and core principles

The treatment of duodenal ulcer perforation follows a stepwise progression, centered on individualized selection spanning conservative management, minimally invasive procedures, and open surgery. Its treatment modalities, complications, and core principles are summarized as follows:

Non-surgical management is strictly reserved for acute small perforations with spontaneous sealing and no diffuse peritonitis; however, disease progression (e.g., abscess formation) is associated with a 6.2% mortality rate, necessitating close monitoring (9). Endoscopic stenting serves as a minimally invasive alternative for elderly patients with multiple comorbidities to avoid surgical trauma, but it carries complications such as stent migration, obstruction, and implantation failure (requiring conversion to surgery), with efficacy yet to be fully validated (10). Laparoscopic surgery has a conversion rate to open surgery of approximately 16%, primarily due to large perforation size, deep location, or suboptimal surgical field visualization; nonetheless, it significantly reduces infection risk and shortens hospital stay (mean 2.6 days) compared to open surgery. Its efficacy is highly surgeon-dependent, with insufficient experience increasing the risk of suture failure (11).

In open surgery, simple suture repair is the preferred approach. High-risk cases (e.g., large perforations, tissue edema, chronic ulcers) are prone to suture failure, fistula formation, or peritonitis (requiring reoperation) (12, 13). Postoperative recurrence rates reach 57.4% in patients with chronic ulcers (low recurrence for acute perforations); infectious complications (e.g., intra-abdominal abscess, surgical site infection) and an overall mortality rate of 6.3% are common, with higher risks in elderly patients, those with comorbidities, or those with delayed surgery. Radical procedures such as vagotomy have a lower mortality rate (2.8%) but are associated with increased complications including delayed gastric emptying and anastomotic fistula, requiring cautious use in high-risk patients (e.g., shock, perforation duration >24 h) (14, 15).

Core treatment principles include: (1) All perforations require urgent intervention, as delayed surgery significantly increases mortality (16); (2) Simple suture repair or laparoscopic suturing is preferred for small perforations/acute ulcers, while radical surgery, stenting (for high-risk patients), or simple suture repair is selected based on tissue conditions for large perforations/chronic ulcers; surgery should be simplified to save lives in cases of septic shock (17); (3) Given that 90% of perforations are associated with *Helicobacter pylori* (Hp), postoperative triple therapy for Hp eradication is required to prevent recurrence (18); (4) Laparoscopy should be the first-line option when technically feasible (especially in young patients), while stenting is only recommended for high-risk surgical candidates (19); and (5) Long-term proton pump inhibitor (PPI) therapy for acid suppression and recurrence monitoring is necessary for patients with chronic ulcer perforation (19).

### Key clinical features and pathophysiological insights of the case

This case is characterized by a paraplegic patient with non-functional right kidney, perirenal abscess, and postoperative duodenal perforation, involving progressive management of complex multisystem complications. The clinical course, spanning March to July 2025, provides critical insights into the treatment of severe infections and postoperative complications in special populations.

### Pathogenesis of infection and exclusion of tuberculosis

The formation of perirenal abscess and fever was driven by complex bacterial infection resulting from the superimposition of multiple high-risk factors. Long-term paraplegia caused local blood supply disorders and neuroregulatory abnormalities, which, combined with immunosuppression from type 2 diabetes (preoperative blood glucose: 7.94 mmol/L, elevated), predisposed the patient to infection. Urinary stasis due to right renal non-function further facilitated bacterial colonization. Preoperative evaluation on March 23, 2025, revealed purulent drainage from the retroperitoneal tube, indicating retroperitoneal space involvement (Figure 3) (20).

### Stepwise management of neurogenic gastrointestinal dysfunction and duodenal perforation

The patient had pre-existing neurogenic gastrointestinal dysfunction, which had been formally diagnosed as gastroparesis syndrome/duodenal stasis (March 26, 2025).

Duodenal perforation, a severe postoperative complication following surgery on March 21, was managed in accordance with the “conservative-minimally invasive-surgical” stepwise principle and individualized approach. Persistent coffee-ground fluid drainage from the retroperitoneum 25 days postoperatively (April 15) led to endoscopic diagnosis of a 4 mm post-bulbar perforation. Endoscopic clipping was prioritized as a minimally invasive option, suitable for the patient’s high-risk profile (paraplegia + diabetes) to reduce surgical trauma, though it carried the risk of fistula non-healing (especially in chronic ulcers or poor tissue conditions). Due to persistent leakage after clipping, radical surgery (subtotal gastrectomy + Roux-en-Y gastrojejunostomy + cholecystolithotomy) was performed. Following discussion with the patient and family, simple suture repair was declined due to a 57.4% risk of recurrence in chronic ulcers and compromised local tissue quality. Laparoscopic repair was precluded by the depth of the perforation and the presence of dense inflammatory adhesions from prior infection. After endoscopic clipping failed, intraoperative findings necessitated a radical approach: the perforation site exhibited extensive friability and induration, precluding any attempt at local repair; it was precisely located at the duodenal bulb adjacent to the pancreas, with anatomy severely distorted by previous infection, rendering local resection technically unfeasible; and underlying chronic duodenal stasis posed an unacceptably high risk of fistula formation with simple drainage. The patient and family ultimately opted for radical resection—subtotal gastrectomy with Roux-en-Y reconstruction—to definitively exclude the diseased segment and ensure secure healing in this high-risk individual (Figure 2) (11–13, 21, 22). This perforation likely resulted from iatrogenic injury during adhesiolysis of densely inflamed tissue, compounded by neurogenic dysmotility increasing intraluminal pressure and impaired diabetic healing. Initially contained, it progressed to complete perforation by day 6—highlighting vigilance for occult injury in high-risk patients despite intraoperative integrity.

To prevent corrosion of major blood vessels by gastrointestinal fluids and pancreatic enzymes, an additional double-lumen tube was placed in the retroperitoneal space to enable countercurrent irrigation and drainage. This intervention served to dilute digestive fluids, thereby mitigating the risk of major vascular bleeding. Notably, computed tomography (CT) imaging confirmed that the retroperitoneal extravasated digestive fluid was confined within a localized encapsulated compartment—a critical anatomical and pathological feature that prevented the progression to severe peritoneal infection and the development of typical signs of peritonitis (Figure 1) (23).

### Nutritional support was provided throughout the treatment course

Based on a high-risk nutritional assessment (NRS score: 5, SGA grade: B) on March 23, a “total parenteral nutrition (TPN) to enteral nutrition transition” model was adopted: initial TPN with amino acids, fat emulsion, and glucose providing 2000 kcal/day of energy and 80 g/day of protein, followed by gradual transition to enteral nutrition starting at 50 mL/h from April 17. Collaborating with the endocrine department, glycemic control was achieved with an insulin pump, laying the foundation for tissue repair (Figure 4) (24).

### Value of multidisciplinary team collaboration and medical spirit

The successful treatment of this case highlights the medical spirit of “never giving up” and the irreplaceable value of MDT collaboration. The patient had 5 high-risk factors (paraplegia, type 2 diabetes, multidrug-resistant infection, recurrent sinus tract, and postoperative perforation) and was refused treatment by multiple tertiary hospitals. However, our team initiated the MDT mechanism on March 23, conducting consultations with departments including urology, infectious diseases, nutrition, and burn surgery (25).

Key decisions led by the MDT included: (1) During the initial surgery on March 21, combined with the burn department to precisely resect the non-functional kidney and abscess, along with debridement of the lumbodorsal sinus tract; (2) Emergency surgical intervention within 1 h of confirming perforation on April 15 to prevent septic shock; and (3) During infection control, the antibiotic regimen was adjusted three times based on drug susceptibility results (switched to cefoperazone-sulbactam on April 3 → vancomycin on April 15 → imipenem 1.0 g via intravenous drip every 8 h combined with caspofungin on April 20). The administration scheme was based on pharmacokinetic/pharmacodynamic (PK/PD) principles, targeting the treatment of multidrug-resistant bacteria and fungal mixed infections (Figure 4) (26).

By late July 2025, all drainage tubes were removed, inflammatory markers returned to normal, sacral grade II pressure ulcers healed with recombinant human epidermal growth factor and mupirocin ointment, and blood glucose was stabilized, achieving clinical cure. This process demonstrates the responsibility of clinicians in managing complex conditions and validates the role of MDT collaboration in addressing multisystem critical illnesses, providing a replicable clinical pathway for similar high-risk patients (Figure 4).

### Limitations of the study

Despite achieving clinical cure through multidisciplinary collaboration and staged management, this study has three principal limitations. First, experience with individualized optimization of retroperitoneal double-lumen drainage was insufficient: parameter selection—including catheter placement, irrigation rate (20 mL/h normal saline), and suction pressure (−8 kPa)—relied on routine practice without validation against the unique retroperitoneal anatomy of paraplegic patients or the characteristics of digestive fluid leakage. Neither dynamic monitoring of pancreatic enzyme activity in drainage fluid nor imaging-guided tube adjustment was performed, risking suboptimal irrigation or excessive tissue edema. Second, infection monitoring lacked timeliness: early postoperative reliance on conventional inflammatory markers rather than metagenomic next-generation sequencing delayed detection of *Enterococcus faecium* colonization until April 9 and missed early warning signs of *Candida albicans* infection, which was only confirmed in late April. Third, intraoperative vigilance for occult injury was insufficient—a critical retrospective learning point. During the initial nephrectomy, despite apparently intact dissection planes, the dense inflammatory adhesions between the duodenum, colon, and right kidney warranted routine integrity testing of adjacent viscera. A simple intraoperative maneuver—instillation of methylene blue or air via nasogastric tube to assess for retroperitoneal leakage—should have been performed to exclude gastrointestinal injury. The omission of such testing denotes a missed opportunity for prompt detection and repair, and underscores that in future complex adhesiolysis cases, intraoperative integrity testing of adjacent viscera should be standard practice prior to closure to prevent severe delayed complications such as the perforation observed here.

## Conclusion

We report a case of postoperative duodenal perforation following right nephrectomy in a high-risk patient with paraplegia and diabetes. Early diagnosis using computed tomography (CT) and endoscopy, targeted surgery guided by MDT collaboration, and 3 months of nutritional support and metabolic regulation contributed to the patient’s successful recovery. This study emphasizes the need for vigilance regarding occult gastrointestinal complications in high-risk surgical patients and confirms that early precise diagnosis and multidisciplinary collaborative intervention are critical to improving prognosis.

## Data Availability

The original contributions presented in the study are included in the article/Supplementary material, further inquiries can be directed to the corresponding author.

## Supplementary material

SUPPLEMENTARY FIGURE 1 Volume changes of drainage fluid from different drainage tubes. Abscissa: postoperative time points; Ordinate: drainage volume (mL). Curves correspond to urinary catheter, cholecystostomy tube, retroperitoneal drainage tube. Peak retroperitoneal volume (1770 mL, April 14–15) aligns with duodenal fistula leakage; subsequent decrease indicates complication control. Urinary/cholecystostomy volumes remain normal, reflecting stable organ function.

SUPPLEMENTARY FIGURE 2 Timeline of key clinical events. The chronology spans from the initial spinal cord injury (2019) to the final follow-up (July 2025), highlighting major diagnoses, interventions, and complications.
