# Supplementary figures and images for: Duodenal perforation following right nephrectomy in a paraplegic patient with pyonephrosis: a case report and clinical insights
Source: Front Med (Lausanne). 2026 Apr 1;13:1745528. doi: 10.3389/fmed.2026.1745528 (PMC13079055; doi:10.3389/fmed.2026.1745528)

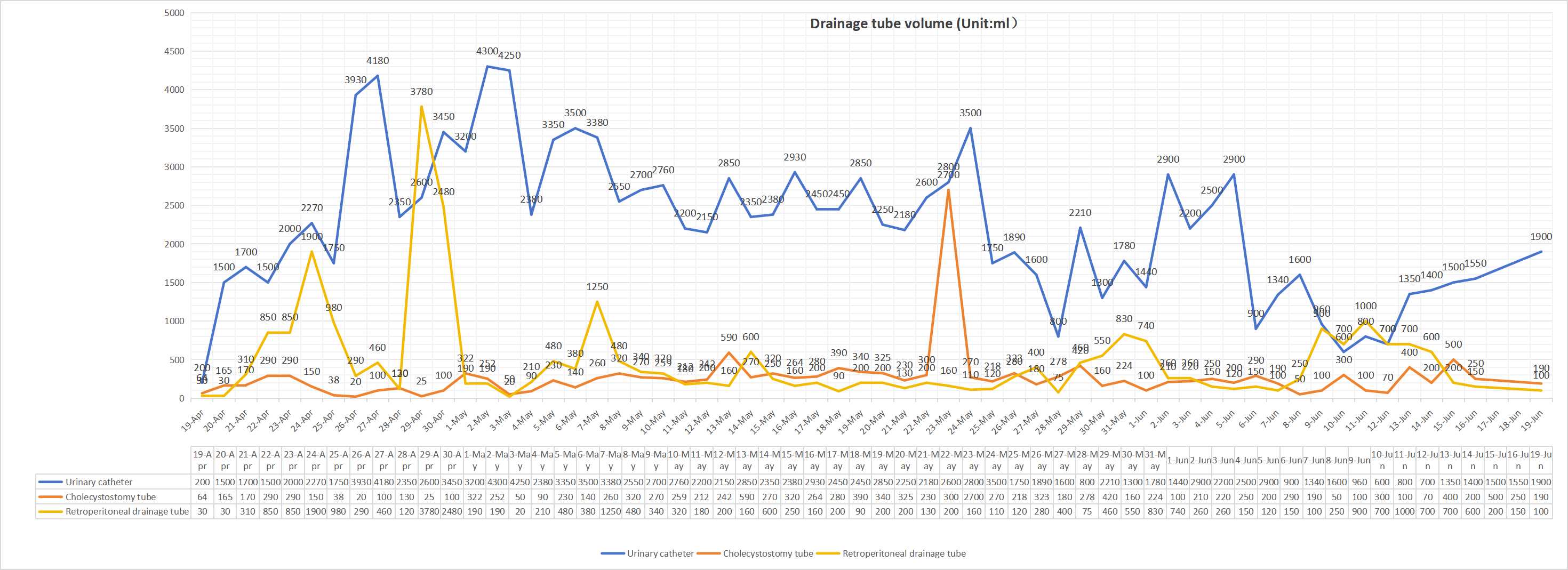

Supplement: SUPPLEMENTARY FIGURE 1 — Volume changes of drainage fluid from different drainage tubes. Abscissa: postoperative time points; Ordinate: drainage volume (mL). Curves correspond to urinary catheter, cholecystostomy tube, retroperitoneal drainage tube. Peak retroperitoneal volume (1770 mL, April 14–15) aligns with duodenal fistula leakage; subsequent decrease indicates complication control. Urinary/cholecystostomy volumes remain normal, reflecting stable organ function. [file Image_1.jpeg]

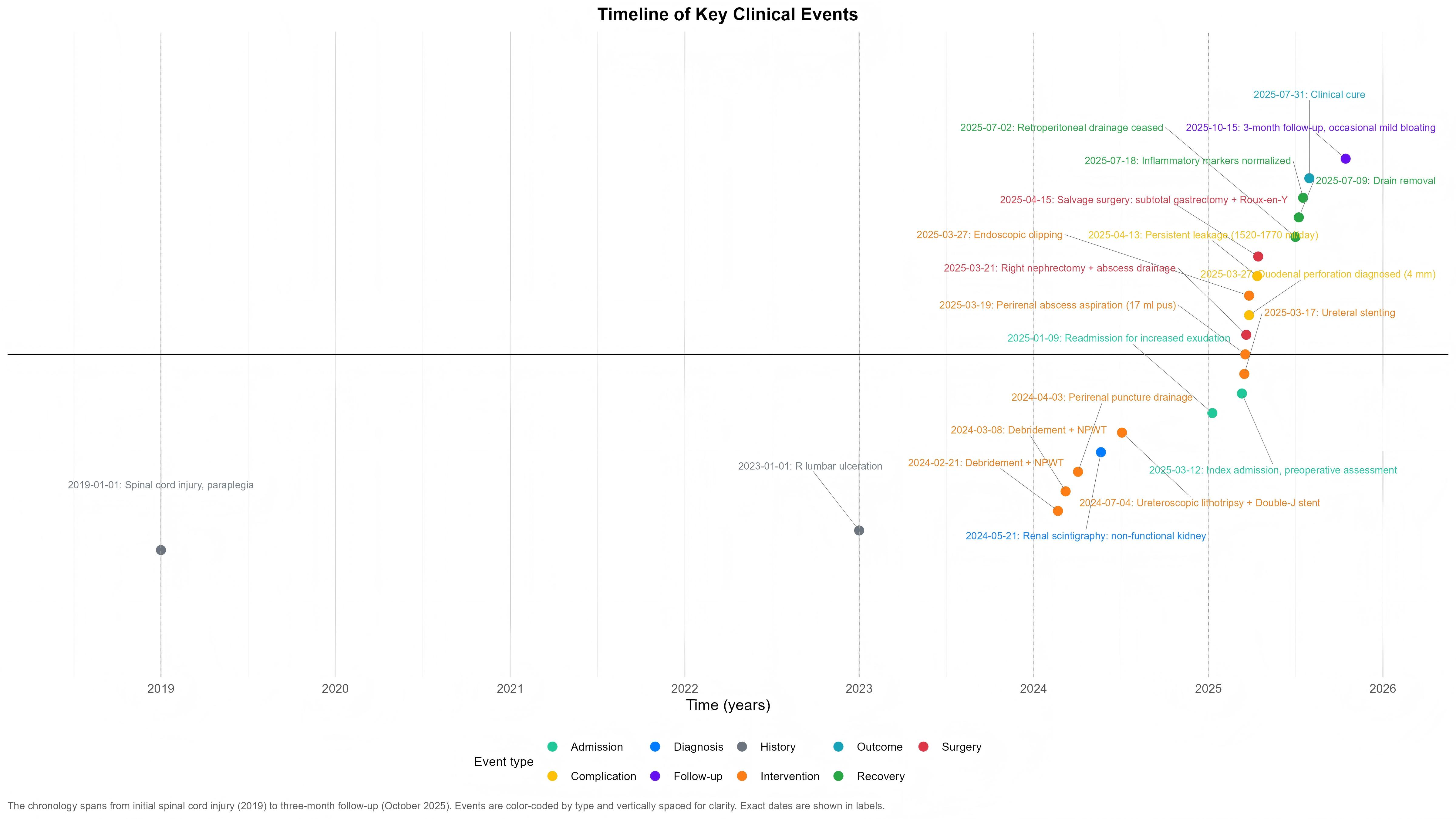

Supplement: SUPPLEMENTARY FIGURE 2 — Timeline of key clinical events. The chronology spans from the initial spinal cord injury (2019) to the final follow-up (July 2025), highlighting major diagnoses, interventions, and complications. [file Image_2.jpeg]
